# The association of cell adhesion molecules and selectins (VCAM-1, ICAM-1, E-selectin, L-selectin, and P-selectin) with microvascular complications in patients with type 2 diabetes: A follow-up study

**DOI:** 10.3389/fendo.2023.1072288

**Published:** 2023-02-09

**Authors:** Khalid Siddiqui, Teena P. George, Muhammad Mujammami, Arthur Isnani, Assim A. Alfadda

**Affiliations:** ^1^ Strategic Center for Diabetes Research, College of Medicine, King Saud University, Riyadh, Saudi Arabia; ^2^ University Diabetes Center, King Saud University Medical City, King Saud University, Riyadh, Saudi Arabia; ^3^ Department of Medicine, College of Medicine, and King Saud University Medical City, King Saud University, Riyadh, Saudi Arabia; ^4^ Obesity Research Center, College of Medicine, King Saud University, Riyadh, Saudi Arabia

**Keywords:** adhesion molecule, diabetic neuropathy, diabetic retinopathy, diabetic nephropathy, complications

## Abstract

**Objective:**

Chronic hyperglycemia induces pathogenic changes in the vascular endothelium and leads to the development of microvascular complications in patients with type 2 diabetes mellitus. Early identification of markers of diabetes complications may help to minimize the risk of the development and progression of microvascular complications.

**Methods:**

This follow-up study was conducted in type 2 diabetic cohort aged between 30-70 years. Out of 160 eligible participants, 70 of them completed follow-up. Levels of cell adhesion molecules and selectins (VCAM-1, ICAM-1, E-selectin, L-selectin and P-selectin) at baseline and follow-up were measured using Randox Evidence biochip analyzer (UK). Development of microvascular complications (diabetic neuropathy, retinopathy and nephropathy) was evaluated.

**Results:**

During the follow-up (2 years, median), 31 (44.3%) developed diabetic neuropathy, 10 (14.3%) developed diabetic retinopathy and, 27 (38.6%) developed diabetic nephropathy. A significant difference in levels of cell adhesion molecules and selectins were found in type 2 diabetic patients with and without microvascular complications. Multiple logistic regression analysis reveals that baseline level of VCAM-1 is significantly associated with microvascular complications; diabetic neuropathy(p=0.028), retinopathy (p=0.007) and nephropathy(p=<0.001). Additionally, levels of P-selectin (p=0.05) and L-selectin (p=0.008) is associated with diabetic nephropathy while retinopathy associated with L-selectin (p=0.005) only.

**Conclusion:**

Cell adhesion molecules and selectins are indicators of microvascular complication among patients with type 2 diabetes (T2D). Association of these markers with the development of microvascular complications may provide additive information for developing strategies for diabetes management and prediction of microvascular complications.

## Introduction

Diabetes mellitus is a chronic metabolic disorder associated with global health issues of the 21^st^ century. As estimated by International Diabetes Federation (IDF), 463 million adults aged 20-79 years worldwide have diabetes in 2019, and this number is projected to reach 578 million by 2030 (1). Globally, type 2 diabetes mellitus is the most common endocrine disorder and its prevalence rapidly growing due to population aging, economic development and increasing urbanization leading to sedentary lifestyle modifications and unhealthy food habits linked with obesity. Chronic hyperglycemia induces pathogenic changes in the vascular wall and increases the incidence of micro and macrovascular complications associated morbidity and mortality among patients with diabetes mellitus. United Kingdom Prospective Diabetes Study (UKPDS), an earlier landmark multi-center trial, have increased the understanding of diabetes management and its beneficial impact on the occurrence of vascular complications, which in turn improve the quality of life and longevity of patients with diabetes (2). Recently, a meta-analysis further clarifies that intensive glucose control could reduce microvascular events (3). Although glycemic control reduces the development and progression of microvascular events, the morbidity associated with these complications are still alarming. Achieving optimal glycemic control on a long-term basis is required to prevent or delay the development and progression of diabetic vascular complications. In addition, early identification of markers of diabetes complications may help to minimize the risk of development and progression of vascular complications as well as the rate of incidence.

Development of vascular complications in patients with diabetes is complex and multifactorial. Even though the precise mechanism is not yet fully elucidated, hyperglycemia induces glucotoxicity, lipotoxicity associated with hyperlipidemia, originated from obesity and insulin resistance seems to be strongly related. Oxidative stress results from hyperglycemia and release of pro-inflammatory cytokines by the adipose tissue leads to low grade inflammation and endothelial dysfunction (4). Additionally, longer duration of diabetes mellitus eventually induces systemic endothelial dysfunction and chronic inflammation leading to microvascular complications such as diabetic neuropathy, diabetic retinopathy and diabetic nephropathy (5).

The adhesion of leucocytes to endothelial cells is an early critical step in the development of vascular complications. These molecules mediate inflammation, endothelial dysfunction and development of micro and macrovascular complications through sequential steps controlled by specific adhesion molecules on leucocytes and endothelial cells. Major cell adhesion molecules involved in the development of microvascular complications are vascular cell adhesion molecule-1 (VCAM-1), intercellular adhesion molecule-1 (ICAM-1), and selectins (E-selectin, L-selectin and P-selectin) (6, 7). Evidences show that levels of cell adhesion molecules were altered in type 2 diabetic patients with microvascular complications including neuropathy, retinopathy and nephropathy (6, 7). Moreover, early detection of altered levels of these molecules in the circulation will predict the development of microvascular complications (8). Due to the complexity of mechanisms involved in the disease pathology, much more effort is needed to efficiently control and manage diabetes mellitus and its complications. However, most clinical trials assessed the levels of cell adhesion molecules and incidence of each microvascular complications exclusively (6, 8). Meanwhile, this study assess the levels of both cell adhesion molecules and selectins, and development of all diabetic microvascular complications in a single study. This follow-up study is performed in patients with T2D to investigate the difference in levels of cell adhesion molecules and selectins among patients with and without microvascular complication and its role in the development of microvascular complications including diabetic neuropathy, diabetic retinopathy and diabetic nephropathy over a two-year period.

## Methods

### Study design

**Figure 1:**
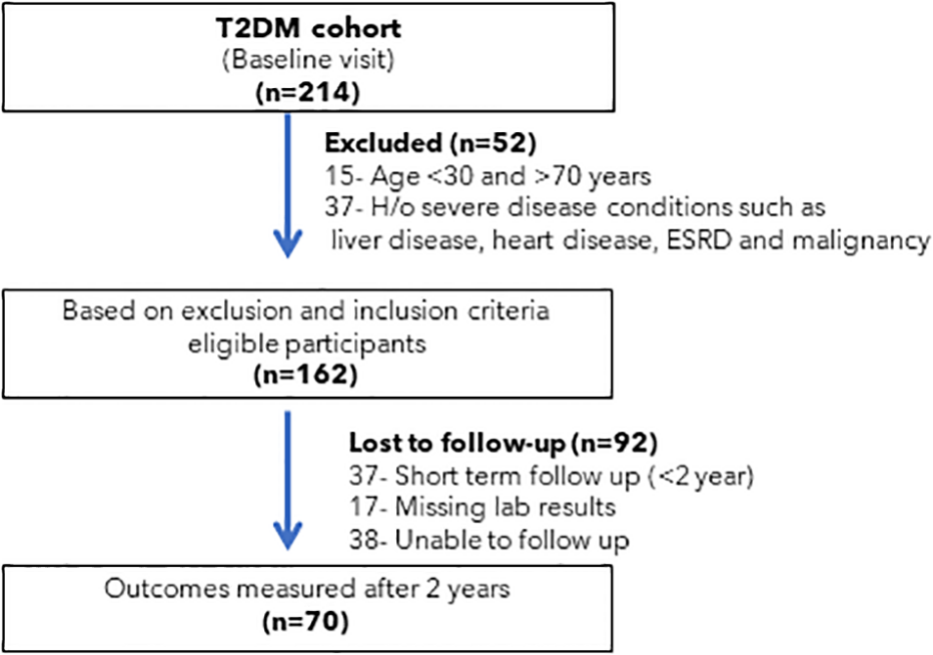
Shows the selection and recruitment of participants.

This study was conducted in type 2 diabetic cohort who followed up in the out-patient clinic of University Diabetes Center (UDC), King Saud University Medical City (KSUMC), Riyadh, Saudi Arabia. Two hundred and fourteen type 2 diabetic patients were selected for this study. Then, excluded patients if they met the following criteria: (1) age <30 and >70 years; (2) patients with history of severe disease condition such as end-stage renal disease, heart failure, liver dysfunction, malignancy (3) and pregnant women. After applying exclusion criteria, 162 patients with type 2 diabetes were selected for follow-up. After 2 years, patients’ data were collected, level of markers and development of microvascular complications was evaluated. Patients who were not regularly followed-up in the clinic over the 2-year period was excluded. After all inclusion and exclusion criteria 70 patients fulfill the criteria for further evaluation. Selection and recruitment of participants were shown in **Figure 1**. Diagnosis of the diabetes mellitus was based on ADA criteria (9). All medications data was recorded. Diabetes management was mainly based on metformin and/or insulin (baseline; metformin (85.8%) and insulin (69.8%), follow-up; metformin (82.9%) and insulin (87.1%). The use of angiotensin receptor blocker was 35.8% (baseline) and 38.6% (follow-up). Among follow-up participants, 30.4% and 15.9% followed dietary recommendations and exercise respectively to manage diabetes mellitus. 97.1% of the participants were non-smokers. This study is approved by Institutional Review Board (IRB), College of Medicine, King Saud University, KSA. An informed consent was obtained from all subjects and/or their legal guardian(s) as the approved study by institute ethics board. This study is according to the guidelines of Strengthening the Reporting of Observation Studies in Epidemiology (STROBE) for cohort studies (10).

### Outcome measures

Three outcomes were included. Diabetic nephropathy was estimated based on KDIGO (Kidney Disease Improving Global Outcomes) guidelines (11). CKD-EPI creatinine equation was used to calculate eGFR (12). Presence of diabetic neuropathy was detected using nerve conduction velocity of upper and lower extremities. Diabetic retinopathy among participants were diagnosed based on the presence of at least one definite microaneurysm in any photographed field and a grading level of > or equal to 20 was considered (13). Diabetic retinopathy and neuropathy were diagnosed by experienced doctors.

### Biochemical measurement

Levels of cell adhesion molecules at baseline were measured from the stored samples at -80^°^C. Randox Evidence biochip analyzer (UK) was used to measure the levels of cell adhesion molecules and selectins (VCAM-1, ICAM-1, E-selectin, L-selectin and P-selectin). Biochemical parameters such as fasting blood glucose (FBG), total cholesterol, triglyceride, high-density lipoprotein (HDL) and low-density lipoprotein (LDL), HbA1c, urea and creatinine were analyzed using RX Daytona clinical chemistry analyzer, Randox, UK.

### Statistical analysis

All statistical analyses were performed using the Statistical Package for Social Sciences software (SPSS) version 23.0 (SPSS-IBM Inc., Armonk, New York, USA). The baseline characteristics were expressed as mean ± standard deviation (SD) values and median (interquartile range) for continuous variables and frequencies with percentages for categorical variables. The statistical significance of differences in continuous variables between two groups were determined through the independent samples t-test. The Chi-square test was performed to compare categorical variables. Percentage distribution of participants in different groups according to the DM duration, hypertension and quartiles of HbA1c was shown by stacked bar chart plotted using MS office excel (2016). Multivariable logistic regression and binary logistic regression were done for diabetic nephropathy, retinopathy and neuropathy. Models were constructed to include VCAM-1, ICAM-1, E-selectin, P-selectin and L-selectin and adjustments were made for age, sex, diabetes mellitus duration, systolic blood pressure, diastolic blood pressure, HbA1c, BMI, LDL cholesterol and eGFR (diabetic nephropathy group). OR with 95% CI and p values were generated to determine the most significant factors for diabetic nephropathy, retinopathy and neuropathy. Receiver Operator Characteristics (ROC) analysis was performed for adhesion molecules and selectins to determine the sensitivity and specificity of these molecules for diabetic neuropathy, diabetic retinopathy and diabetic nephropathy. P value of <0.05 was considered statistically significant.

## Results

A total of 162 patients with T2D were selected at baseline with a median age of 55 years, and 45.7% were male. Median duration of diabetes was 19.0 years. Patients were either overweight or obese (mean BMI 33.1 ± 6.0 kg/m^2^) with poor glycemic control (mean HbA1c 9.2 ± 2.03%). Among 162, seventy patients (43.2%) came for follow-up. The mean BMI was 34.5 kg/m^2^ (SD 5.9) and mean HbA1c was 10.2% (SD 1.5). Level of cell adhesion molecules and selectins were shown in **Table 1**.

**Table 1:** Demographic and biochemical characteristics of selected study participants at baseline and after 2 years of time period.

Parameters	Baseline (n=162)	Follow-up (n=70)
Age (years)	54.5 ± 8.2	55.1 ± 6.8
DM duration (years)	18.7 ± 6.6	20.9 ± 5.5
Systolic blood pressure (mm Hg)	132.8 ± 15.4	134.2 ± 14.58
Diastolic blood pressure (mm Hg)	73.2 ± 8.8	69.2 ± 9.4
BMI (kg/m^2^)	33.1 ± 6.0	34.5 ± 5.9
Waist circumference (cm)	105.0 ± 11.9	110.2 ± 11.9
Waist to hip ratio	0.94 ± 0.07	0.97 ± 0.0
Total cholesterol (mmol/l)	4.3 ± 0.9	4.77 ± 1.0
Triglyceride (mmol/l)	1.7 ± 0.9	2.1 ± 1.0
HDL cholesterol (mmol/l)	1.2 ± 0.6	1.2 ± 0.2
LDL cholesterol (mmol/l)	2.4 ± 0.7	3.5 ± 1.0
FBG (mmol/l)	9.3 ± 3.7	11.6 ± 4.9
HbA1c (%)	9.2 ± 2.03	10.2 ± 1.5
Urea (mmol/l)	5.7 ± 3.0	15.1 ± 9.3
Creatinine (μmol/l)	80.6 ± 38.9	112.3 ± 73.4
eGFR (ml/min/1.73m^2^)	87.5 ± 23.5	68.7 ± 38.9
Metformin (%)	85.8	82.9
Insulin (%)	69.8	87.1
Angiotensin receptor blockers (%)	35.8	38.6
VCAM-1, ng/ml [median (IQR)]	566.6 (429.1–718.9)	901.7 (675.8–1179.8)
ICAM-1, ng/ml [median (IQR)]	283.2 (237.2–340.7)	344.3 (279.5–391.7)
E-selectin, ng/ml [median (IQR)]	22.5 (16.7–30.5)	31.4 (21.7–40.1)
P-selectin, ng/ml [median (IQR)]	192.7 (164.1–238.7)	250.2 (202.8–308.2)
L-selectin, ng/ml [median (IQR)]	1371.6 (1122.5–1631)	1801.6 (1495.1–2176.9)

Data represents in mean ± standard deviation and median (interquartile range). DM duration (diabetes mellitus duration), BMI (body mass index), HDL cholesterol (high density lipoprotein cholesterol), LDL cholesterol (low density lipoprotein cholesterol), FBG (fasting blood glucose), VCAM-1 (vascular cell adhesion molecule-1), ICAM-1 (intercellular adhesion molecule-1).

**Figure 2:**
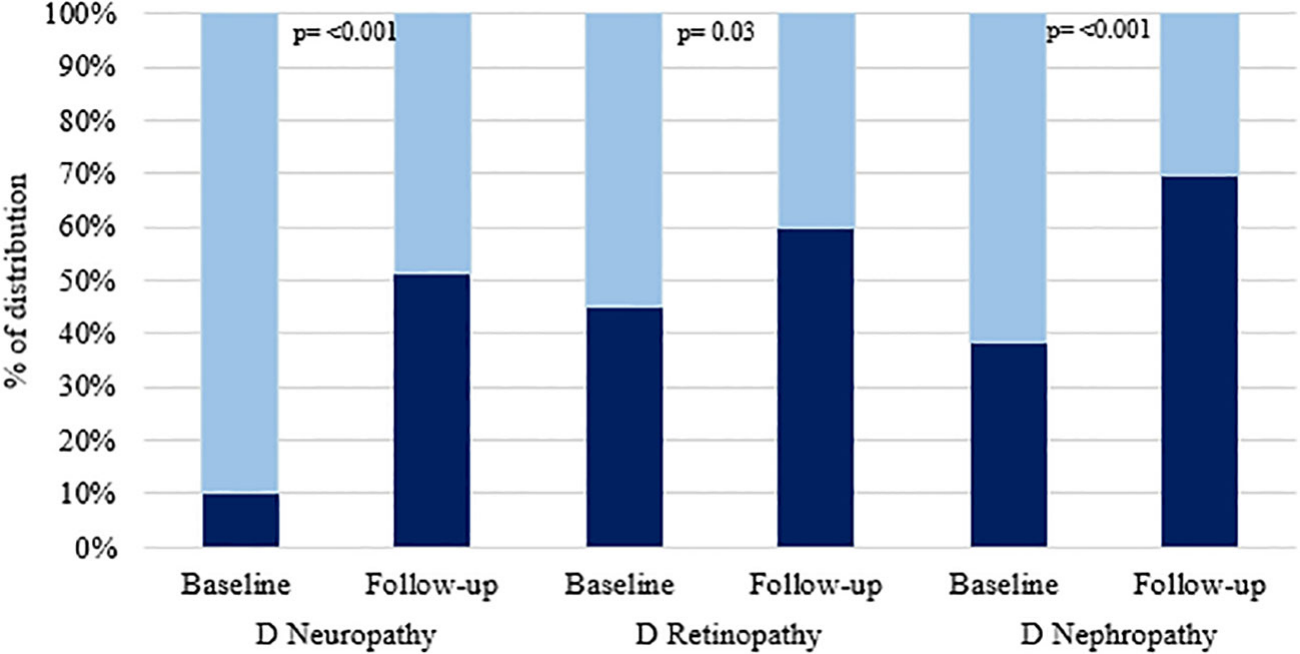
Shows the percentage of distribution of microvascular complications among type 2 diabetic patients at baseline (N=162) and follow-up (N=70). Dark color shows the “presence” and light color shows “absence” of corresponding complication.

During the median follow-up period of two years, among 70 patients with T2D, 31 (44.3%) developed diabetic neuropathy, 10 (14.3%) developed diabetic retinopathy and, 27 (38.6%) developed diabetic nephropathy and 20 (28.6%) developed any of the microvascular complications (**Supplementary Table 1**). Percentage distribution of microvascular complications among patients with diabetes at baseline and follow-up is shown in **Figure 2**. After two years of follow-up period, the number of participants who developed diabetic neuropathy, diabetic retinopathy and diabetic nephropathy were significantly increased. **Table 2** shows the difference in levels of cell adhesion molecules and selectins in patients with and without microvascular complications. The level of cell adhesion markers and selectins were significantly differed between type 2 diabetes patients without any complications at baseline with those have diabetic neuropathy [VCAM-1 (p=<0.001) and L-selectin (p=0.03)]; diabetic retinopathy [VCAM-1 (p=<0.001), ICAM-1 (p=0.01) and L-selectin (p=0.04)]; diabetic nephropathy [VCAM-1 (p=<0.001), ICAM-1 (p=0.008), P-selectin(p=0.02) and L-selectin (p=0.009)] and patients with any of the microvascular complication [VCAM-1 (p=<0.001), ICAM-1 (p=0.01), P-selectin (p=0.04) and L-selectin (p=0.007)] at follow-up. In addition, the level of cell adhesion molecules and selectins were found to be significantly higher among those who develop different microvascular complications namely, diabetic neuropathy (VCAM-1 p=0.002; E-selectin p=0.04; P-selectin p=0.01; L-selectin p=0.003), diabetic retinopathy (VCAM-1 p= 0.008; L-selectin p=0.007), diabetic nephropathy (VCAM-1 p=<0.001; ICAM-1 p=0.006; E-selectin p=0.03; P-selectin p=0.001; L-selectin p=0.01) and any of the microvascular complication group (VCAM-1 p=0.002; L-selectin p=0.02) when compared with their level at baseline (**Supplementary Table 1**).

**Table 2:** Comparison of the level of cell adhesion molecules and selectins in different categories of microvascular complications^#^.

Marker	T2D without any complication (Baseline)	Diabetic neuropathy (Follow-up)	Diabetic retinopathy (Follow-up)	Diabetic nephropathy (Follow-up)	Microvascular complications (Follow-up)
Median (IQR)	(n=27)	Yes (n=36)	Yes (n=42)	Yes (n=49)	Yes (n=63)
VCAM-1, ng/ml	464.87 (371.4 ± 723.25)	905.75 (652.84 ± 1221.25) ^**^	879.23 (669.58 ± 1179.80) ^**^	926.72 (688.59 ± 1184.28) ^**^	897.33 (670.40 ± 1178.32) ^**^
ICAM-1, ng/ml	279.47 (196.21 ± 344.02)	335.52 (237.73 ± 393.55)	347.58 (279.59 ± 384.37) ^*^	337.04 (284.20 ± 389.36) ^**^	337.04 (277.44 ± 390.66) ^*^
E-selectin, ng/ml	26.58 (20.23 ± 37.89)	29.88 (20.19 ± 39.11)	33.40 (22.55 ± 40.32)	31.83 (21.66 ± 39.30)	30.54 (21.53 ± 39.28)
P-selectin, ng/ml	204.57 (170.79 ± 244.08)	231.59 (166.29 ± 293.86)	243.38 (180.91 ± 305.63)	250.95 (204.88 ± 325.14) ^*^	244.22 (197.44 ± 305.20) ^*^
L-selectin, ng/ml	1541.08 (1006.08 ± 1969.42)	1720.18 (1435.36 ± 1997.85) ^*^	1765.42 (1402.86 ± 2098.12) ^*^	1791.08 (1480.40 ± 2149.96) ^**^	1791.08 (1458.10 ± 2146.04) ^**^

Data represents in median (interquartile range). VCAM-1 (vascular cell adhesion molecule-1), ICAM-1 (intercellular adhesion molecule-1), T2D (type 2 diabetes). Compared between T2D without any complication vs microvascular complications (Yes); T2D without any complication vs diabetic neuropathy (Yes); T2D without any complication vs diabetic retinopathy (Yes); T2D without any complication vs diabetic nephropathy (Yes). *p-value <0.05; ** p-value <0.01. p value <0.05 is statistically significant. (**^#^** comparison of the level of markers of same patients at baseline and follow-up were shown in **Supplementary Table 1**).

Multiple logistic regression was performed to determine the association between cell adhesion molecules and selectins with microvascular complications in patients with type 2 diabetes mellitus after adjusting for age, sex, diabetes mellitus duration, systolic blood pressure, diastolic blood pressure, HbA1c, BMI, LDL cholesterol and eGFR (diabetic nephropathy group). Among the different adhesion molecules, VCAM-1 was the only significantly associated molecule for diabetic neuropathy (OR=0.999, 95%CI=0.997-1.0, p=0.028). For diabetic retinopathy, VCAM-1 and L-selectin were found to be significantly associated with the microvascular complications (OR=0.999, 95%CI=0.997-1.0, p=0.007 and OR=1.0, 95%CI=1.0-1.002, p=0.005, respectively). For diabetic nephropathy, VCAM-1, P-selectin and L-selectin were found to be significantly associated with microvascular complications (OR=0.996, 95%CI=0.995-0.998, p=<0.001, OR=0.995, 95%CI=0.989-1.0, p=0.050, and OR=1.0, 95%CI=1.00-1.002, p=0.008, respectively) (**Table 3**). When cell adhesion molecules and selectins were examined for their association with diabetic microvascular complications using an adjusted binary logistic regression model, serum levels of VCAM-1 [(diabetic neuropathy (OR=0.999, 95% CI=0.997-1.0, p=0.01); diabetic retinopathy (OR=0.999, 95% CI=0.997-1.00, p=0.006); diabetic nephropathy (OR=0.996, 95% CI=0.995-0.998, p=<0.001)], P-selectin (diabetic nephropathy, OR=0.995, 95% CI=0.989-1.00, p=0.02), and L-selectin [diabetic retinopathy (OR=1.00, 95% CI=1.00-1.002, p=0.004); diabetic nephropathy (OR=1.00, 95% CI=1.00-1.002, p=<0.01)] were found to be significantly associated (**Table 4**).

**Table 3:** Multiple logistic regression analysis to determine the association between level of adhesion molecules and selectins with microvascular complications in patients with type 2 diabetes mellitus.

	Diabetic neuropathy		Diabetic retinopathy		Diabetic nephropathy	
	OR (95% CI)	p-value	OR (95% CI)	p-value	OR (95% CI)	p-value
VCAM-1	0.999 (0.997-1.0)	0.028*	0.999 (0.997-1.00)	0.007*	0.996 (0.995-0.998)	<0.001*
ICAM-1	1.01 (0.998-1.01)	0.399	0.998 (0.995-1.00)	0.186	1.0 (0.996-1.004)	0.870
E-selectin	0.994 (0.964-1.024)	0.696	1.01 (0.986-1.04)	0.359	1.02 (0.981-1.05)	0.390
P-selectin	0.998 (0.993-1.003)	0.516	0.998 (0.993-1.00)	0.360	0.995 (0.989-1.0)	0.050*
L-selectin	0.399 (0.999-1.0)	0.399	1.00 (1.00-1.002)	0.005*	1.00 (1.00-1.002)	0.008*

*p value <0.05 is statistically significant.

**Table 4:** Binary logistic regression analysis to determine the association between levels of cell adhesion molecules and selectins with microvascular complications in patients with type 2 diabetes mellitus.

	Diabetic neuropathy		Diabetic retinopathy		Diabetic nephropathy	
	OR (95% CI)	p-value	OR (95% CI)	p-value	OR (95% CI)	p-value
VCAM-1	0.999 (0.997-1.0)	0.018*	0.999 (0.997-1.00)	0.006*	0.996 (0.995-0.998)	<0.001*
ICAM-1	1.01 (0.998-1.01)	0.370	0.998 (0.995-1.00)	0.181	1.0 (0.996-1.004)	0.729
E-selectin	0.994 (0.964-1.024)	0.655	1.01 (0.986-1.04)	0.350	1.02 (0.981-1.05)	0.464
P-selectin	0.998 (0.993-1.003)	0.490	0.998 (0.993-1.00)	0.336	0.995 (0.989-1.0)	0.028*
L-selectin	0.399 (0.999-1.0)	0.368	1.00 (1.00-1.002)	0.004*	1.00 (1.00-1.002)	0.011*

*p value <0.05 is statistically significant.

**Figure 3:**
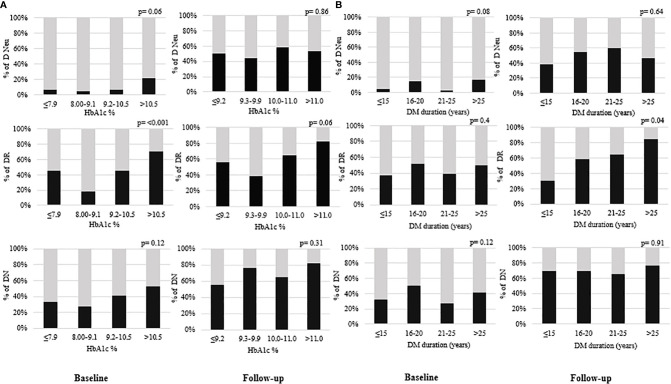
**(A)** shows the percentage distribution of microvascular complications in quartiles of HbA1c and **(B)** shows the percentage distribution of microvascular complications in different groups of DM duration (years) at baseline (N=162) and follow-up (N=70). Dark color shows the “presence” and light color shows “absence” of corresponding complication. DM duration (diabetes mellitus duration), DN (diabetic nephropathy), DR (diabetic retinopathy), D Neu (diabetic neuropathy). P value <0.05 is statistically significant.

To check the effect of diabetes management, we have looked the distribution of different microvascular complications in quartiles of HbA1c at baseline and follow-up time period and shown in **Figure 3A**. At baseline, number of patients with diabetic retinopathy was significantly increased with quartiles of HbA1c. **Figure 3B** shows the percentage distribution of microvascular complications in different groups of DM duration at baseline and follow-up. Number of patients with diabetic retinopathy was increases with increasing duration of diabetes mellitus at follow-up. To assess the involvement of comorbid hypertension, we have looked the percentage distribution of hypertension in different categories of diabetic microvascular complications at baseline and follow-up and shown in **Supplementary Figure 1**. In both baseline and follow-up, number of participants with hypertension was significantly higher among those with diabetic retinopathy.

## Discussion

This study has been conducted to investigate the difference in levels of cell adhesion molecules and selectins in patients with and without microvascular complications and its association with the development of microvascular complications in patients with T2D after 2 years of follow-up. The key findings of the present study are (1) Significant difference in levels of cell adhesion molecules and selectins were found in type 2 diabetic patients with and without microvascular complication. (2) Baseline level of VCAM-1was significantly associated with diabetic neuropathy, retinopathy and nephropathy. Additionally, levels of P-selectin and L-selectin was related with diabetic nephropathy while retinopathy was associated with L-selectin only. (3) Furthermore, number of patients with diabetic retinopathy was significantly increases with quartiles of Hba1c at baseline as well as increasing diabetes duration at follow-up.

Cell adhesion molecules are cell surface proteins, necessary for the cells to adhere to one another and their surroundings and it is essential for tissue formation, cell to cell interaction and cell regulation (14). These molecules mediate blood cell-endothelial cell interaction under physiological and pathological conditions. In patients with diabetes, hyperglycemia induced oxidative stress and release of cytokines leads to the expression of cell adhesion molecules on the activated endothelial cells. These molecules help in the transmigration of leucocytes to the tissues and considered as the mediators between chronic inflammation, endothelial dysfunction and micro and macro vascular complications in patient with diabetes (15–17). Apart from this, many risk factors such as age of patient, hypertension, hyperlipidemia, degree of glycemic control and duration of diabetes mellitus are also identified in the causation of microvascular complications in patients with diabetes mellitus (18).

Prolonged exposure to hyperglycemia is one of the major risk factors for the development and progression of diabetic microvascular complications (2). Poor glycemic control, longer duration of diabetes and hypertension were reported with the occurrence of microvascular complications among patients with type 2 diabetes mellitus (19, 20). In this study, a significant increase in the number of participants with different microvascular complications in the higher quartiles of HbA1c highlight the importance of tight glycemic control in diabetes management. Recently a study of larger population of people with T2D demonstrated longer duration and risk of microvascular complications (21). This study also noticed that number of participants with microvascular complications were found to be more in participants with longer diabetes mellitus duration. Additionally, comorbid hypertension was prevalent among participants with microvascular complications. Lack of physical activity, obesity and hyperlipidemic conditions were also associated with the development of microvascular complications (22, 23). Lack of exercise (83.9%), obesity (71.4%) and hyperlipidemia (90.5%) were noticed among study participants with microvascular complications.

Although multiple cross-sectional studies have demonstrated higher levels of adhesion molecules in diabetic patients with different microvascular complications, limited number of prospective studies have sought to identify these endothelial dysfunction markers linked with the incidence of different microvascular complications (8, 24). Moreover, previous prospective studies were mainly focused on individual microvascular complications, while few cross-sectional studies evaluated the levels of cell adhesion molecules in diabetic microvascular complications (6, 8, 25, 26). It is worth mentioning that this follow-up study assess the levels of both cell adhesion molecules and selectins and included the development of all diabetic microvascular complications in a single study.

In the present study, levels of cell adhesion molecules are found to be increased among those who developed microvascular complications after 2 years of follow-up. VCAM-1 was significantly associated with diabetic neuropathy, retinopathy and nephropathy. Elevated levels of VCAM-1 possibly associated with widespread endothelial activation and dysfunction. In patients with diabetic retinopathy, expression of cell adhesion molecules is critical for adherence of leukocyte into the retinal vessels and transmigrate toward the retina into the sites of inflammation. Based on the previous evidence, increased expression of adhesion molecules and impaired vasodilation observed in retinal micro vessels could leads to increased vessel wall permeability and capillary occlusion in patients with diabetic retinopathy (24, 27, 28). Moreover, poor glycemic control, overweight or obese nature of the studied participants and longer duration of diabetes mellitus further intensify the risk of developing complications. Additionally, a decrease in eGFR was observed in patients who developed diabetic nephropathy. Even though an increase in the level of cell adhesion molecules (ICAM-1 and VCAM-1) and selectins (E-selectin, P-selectin and L-selectin) were found during the development of diabetic nephropathy, a significant association was observed with baseline levels of VCAM-1, P-selectin and L-selectin only. Earlier evidences also show the association between VCAM-1 and diabetic kidney disease and the significant involvement of P-selectin; a marker of platelet activation, which activates procoagulant imbalance in the pathogenesis of diabetic nephropathy among patients with type 2 diabetes mellitus (7, 29, 30). Furthermore, increased renal expression of cell adhesion molecules were observed during the progression of diabetic kidney disease (31, 32). Moreover, a significant increase in the levels of cell adhesion molecules and selectins were found in patients who developed diabetic neuropathy. Studies observed higher level of cell adhesion molecules in patients with diabetic neuropathy than without this complication (8, 33).

Although this study includes patients with major diabetic microvascular complications to evaluate the levels of cell adhesion molecules and selectins in a single study, due to considerable percentage of drop out of patients reduces the sample size. Thus, larger scale clinical trials including various ethnic group is required to further validate the conclusions.

Based on the findings, cell adhesion molecules and selectins are indicators of microvascular complications among patients with T2D. Circulatory levels of cell adhesion molecule (VCAM-1) and selectins (P-selectin and L-selectin) are associated with future development of microvascular complications including diabetic neuropathy, retinopathy and nephropathy. This study also highlights the importance of diabetes management in the development of microvascular complications. Early identification of these markers may well facilitate treatment strategies and reduce the progression of the disease.

## Data availability statement

The original contributions presented in the study are included in the article/**Supplementary Material**. Further inquiries can be directed to the corresponding author.

## Ethics statement

The studies involving human participants were reviewed and approved by Institutional Review Board (IRB), College of Medicine, King Saud University, Saudi Arabia. The patients/participants provided their written informed consent to participate in this study.

## Author contributions

All authors contributed to the study conception and design, acquisition and interpretation of data, critical revision and agreed to be accountable for all aspects of the work. All authors contributed to the article and approved the submitted version.

## Supplementary material

Supplementary Figure 1 Shows the percentage distribution of hypertension in different categories of microvascular complications at baseline (N=162) and follow-up (N=70). DN (diabetic nephropathy), DR (diabetic retinopathy), D Neu (diabetic neuropathy), HTN (hypertension). p value <0.05 is statistically significant.
