# Supplementary material for: The association of cell adhesion molecules and selectins (VCAM-1, ICAM-1, E-selectin, L-selectin, and P-selectin) with microvascular complications in patients with type 2 diabetes: A follow-up study
Source: Front Endocrinol (Lausanne). 2023 Feb 9;14:1072288. doi: 10.3389/fendo.2023.1072288 (PMC9948618; doi:10.3389/fendo.2023.1072288)
Supplement: Supplementary file 1 [file Table_1.docx]

|  |  | | **n** | **VCAM-1** | **p** | **ICAM-1** | **p** | **E-selectin** | **p** | **P-selectin** | **p** | **L-selectin** | **p** |
| --- | --- | --- | --- | --- | --- | --- | --- | --- | --- | --- | --- | --- | --- |
| **Microvascular**  **complications** | Baseline | No |  | 509.5(412.5-711.3) | 0.002 | 282(174.4-343.7) | 0.10 | 24.6(18.2-33.6) | 0.42 | 202(165.9-237) | 0.08 | 1617.8(1054-2008) | 0.02 |
|  | Follow-up | Yes | 20 | 871.7(700.8-1142) |  | 317(268.5-390.6) |  | 29.8(21.8-39.9) |  | 232(202.1-287.0) |  | 1951.5(1682.7-2156) |  |
| **Diabetic neuropathy** | Baseline | No | 31 | 596 (459.7-743.9) |  | 277(255.6-342.8) |  | 23.4(16.7-27.3) |  | 184(162.5-216.6) |  | 1337.7(1198-1694) |  |
|  | Follow-up | Yes |  | 934.9(648-1191) | 0.002 | 329(236.8-404.2) | 0.38 | 29.8(21.1-40.2) | 0.04 | 233(170.6-289.6) | 0.01 | 1753(1426.9-2120) | 0.003 |
| **Diabetic retinopathy** | Baseline | No | 10 | 573(421-725) | 0.008 | 299.7(226-344.2) | 0.1 | 27.4(20.0-33.5) | 0.28 | 205.9(168-238.6) | 0.06 | 1381(1109-1554) | 0.007 |
|  | Follow-up | Yes |  | 866.2(692-1085) |  | 340(298.5-365) |  | 34.5(25.6-42.1) |  | 232(212.5-280) |  | 1998(1600.6-2182) |  |
| **Diabetic nephropathy** | Baseline | No | 27 | 563(423-718) | <0.001 | 291(218.9-351.7) | 0.006 | 25.9(23.4-32.1) | 0.03 | 207(182-244) | 0.001 | 1298(1110-1624) | 0.01 |
|  | Follow-up | Yes |  | 876.5(667-1145.6) |  | 332(290.9-390.6) |  | 35.5(22.6-44.6) |  | 255(219-341.4) |  | 1786(1393-2062) |  |

**Supp.table 1** Comparison of the level of cell adhesion molecules at baseline and after 2 years of follow-up in different categories of microvascular complications

Data represents in median (interquartile range). VCAM-1 (vascular cell adhesion molecule-1), ICAM-1 (intercellular adhesion molecule-1). p value <0.05 is statistically significant.
