# Supplementary material for: The association of cell adhesion molecules and selectins (VCAM-1, ICAM-1, E-selectin, L-selectin, and P-selectin) with microvascular complications in patients with type 2 diabetes: A follow-up study
Source: Front Endocrinol (Lausanne). 2023 Feb 9;14:1072288. doi: 10.3389/fendo.2023.1072288 (PMC9948618; doi:10.3389/fendo.2023.1072288)
Supplement: Supplementary file 2 [file Table_2.docx]

**Supp. table 2** ROC analysis of adhesion molecules and selectins for diabetic neuropathy in patients with type 2 diabetes mellitus

|  | AUC (95% CI) | p-value | Cut-off value | Sensitivity | Specificity |
| --- | --- | --- | --- | --- | --- |
| VCAM-1 | 0.616 (0.517-0.715) | 0.011 | 649 | 60.8% | 54.0% |
| ICAM-1 | 0.559 (0.463-0.655) | 0.198 | 297 | 56.9% | 52.0% |
| E-selectin | 0.602 (0.515-0.690) | 0.026 | 25.4 | 58.8% | 52.0% |
| P-selectin | 0.593 (0.501-0.684) | 0.044 | 216 | 58.8% | 60.0% |
| L-selectin | 0.614 (0.529-0.699) | 0.013 | 1529 | 62.7% | 62.0% |

AUC, area under the receiver operating characteristic curve; CI, confidence interval; p value <0.05 is statistically significant

**Supp. table 3** ROC analysis of adhesion molecules and selectins for diabetic retinopathy in patients with type 2 diabetes mellitus.

|  | AUC (95% CI) | p-value | Cut-off value | Sensitivity | Specificity |
| --- | --- | --- | --- | --- | --- |
| VCAM-1 | 0.619 (0.547-0.692) | 0.002 | 614.2 | 63.2% | 55.3% |
| ICAM-1 | 0.618 (0.545 – 0.690) | 0.002 | 294.2 | 62.3% | 60.0% |
| E-selectin | 0.517 (0.441-0.592) | 0.038 | 22.3 | 61.6% | 45.0% |
| P-selectin | 0.524 (0.449 – 0.599) | 0.038 | 199.0 | 57.1% | 44.0% |
| L-selectin | 0.464 (0.389 – 0.539) | 0.038 | 1390 | 54.5% | 40.0% |

AUC, area under the receiver operating characteristic curve; CI, confidence interval; p value <0.05 is statistically significant

**Supp. table 4** ROC analysis of adhesion molecules and selectins for diabetic nephropathy in patients with type 2 diabetes mellitus

|  | AUC (95% CI) | p-value | Cut-off value | Sensitivity | Specificity |
| --- | --- | --- | --- | --- | --- |
| VCAM-1 | 0.765 (0.696 – 0.834) | <0.001 | 655.6 | 75.4% | 63.0% |
| ICAM-1 | 0.650 (0.570-0.731) | 0.001 | 304.7 | 65.0% | 60.0% |
| E-selectin | 0.553 (0.465-0.641) | 0.231 | 22.6 | 56.1% | 43.0% |
| P-selectin | 0.579 (0.489-0.668) | 0.075 | 206.6 | 59.6% | 50.0% |
| L-selectin | 0.551 (0.465-0.637) | 0.252 | 1437.0 | 54.4% | 54.0% |

AUC, area under the receiver operating characteristic curve; CI, confidence interval; p value <0.05 is statistically significant
