# Supplementary figures and images for: The association of cell adhesion molecules and selectins (VCAM-1, ICAM-1, E-selectin, L-selectin, and P-selectin) with microvascular complications in patients with type 2 diabetes: A follow-up study
Source: Front Endocrinol (Lausanne). 2023 Feb 9;14:1072288. doi: 10.3389/fendo.2023.1072288 (PMC9948618; doi:10.3389/fendo.2023.1072288)

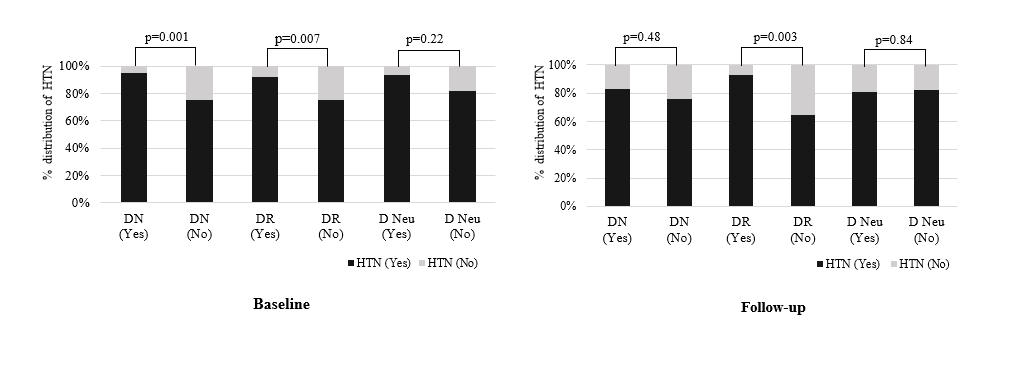

Supplement: Supplementary Figure 1 — Shows the percentage distribution of hypertension in different categories of microvascular complications at baseline (N=162) and follow-up (N=70). DN (diabetic nephropathy), DR (diabetic retinopathy), D Neu (diabetic neuropathy), HTN (hypertension). p value <0.05 is statistically significant. [file Image_1.tif]
